# Microalgae-Mediated Nanotechnology for Sustainable Agriculture: Applications, Advances, and Future Prospects

**DOI:** 10.3390/ijms27135875

**Published:** 2026-06-30

**Authors:** Yu Xie, Zirui Yang, Shoukai Guo, Liqin Sun, Hongli Cui, Zhongliang Sun

**Affiliations:** 1 College of Life Science, Yantai University, Yantai 264006, China; 2 College of Biological Sciences and Technology, University of Jinan, Jinan 250022, China; 3 Laboratory of Coastal Biology and Biological Resource Utilization, Yantai Institute of Coastal Zone Research, Chinese Academy of Sciences, Yantai 264003, China

**Keywords:** microalgae, nanotechnology, green synthesis, sustainable agriculture, nano-fertilizer, antibacterial activity, environmental remediation

## Abstract

The overreliance on chemical pesticides has caused severe environmental contamination, health risks, and increasing pest and pathogen resistance, creating an urgent need for greener and more efficient alternatives in sustainable agriculture. Microalgae-mediated green nano-synthesis has emerged as a promising strategy because of its environmental compatibility, cost-effectiveness, and multifunctional potential. This review critically summarizes recent advances in microalgae-derived nanomaterials for agricultural applications. First, we discuss the biochemical basis of nanoparticle biosynthesis, highlighting the roles of microalgal polysaccharides, proteins, photosynthetic pigments, extracellular polymeric substances, and secondary metabolites as reducing, capping, and stabilizing agents. We then summarize intracellular and extracellular synthesis pathways, advanced synthesis strategies, and key reaction parameters, including temperature, pH, and metal precursor concentration, which regulate nanoparticle size, morphology, stability, and yield. Subsequently, major microalgae-derived nanomaterials, including gold, silver, selenium, zinc oxide, bimetallic, and other functional nanoparticles, are discussed in relation to their agricultural applications. These nanomaterials show potential in bacterial, fungal, and viral disease control, biofilm disruption, plant growth promotion, yield enhancement, and abiotic stress mitigation. Their agronomic effects are associated with multiple mechanisms, including reactive oxygen species generation, pathogen membrane disruption, inhibition of biofilm formation, enhanced nutrient bioavailability, antioxidant regulation, and activation of plant systemic resistance. In addition, this review evaluates the phytotoxicity, biocompatibility, soil microbial impacts, and environmental safety of microalgae-derived nanomaterials, emphasizing that green synthesis does not automatically guarantee biosafety. Finally, we discuss their integration into circular agriculture through CO_2_ capture and wastewater-derived metal recovery, while highlighting remaining challenges in scale-up, quality control, economic feasibility, regulatory classification, and public acceptance. Overall, microalgae-mediated nanotechnology offers a promising platform for developing safer, more efficient, and circular agricultural inputs.

## 1. Introduction

Global population growth continues to place unprecedented pressure on food security, with estimates indicating that agricultural production needs to increase by at least 50% by 2050 to meet projected demands [1]. Chemical pesticides have played a crucial role in modern agriculture over the past decades in maintaining crop yields and protecting against pests and diseases [1,2]. However, their extensive use has resulted in significant environmental and public health challenges. Current estimates suggest that approximately 2 million people worldwide experience pesticide poisoning each year, with approximately 22,000 fatalities [2]. Even more alarming is that over 90% of the applied pesticides fail to reach their target pests and instead contaminate the surrounding environment (e.g., soil, water bodies, and atmosphere) through processes such as runoff, leaching, and atmospheric drift. This environmental contamination by pesticides significantly harms non-target organisms such as pollinating and beneficial insects and aquatic species [2,3]. Moreover, pesticide residues can enter and accumulate in food chains, posing long-term health risks to humans and contributing to chronic diseases such as cancer and neurological disorders [2,4]. In major agricultural river basins such as the Yellow River in China, the excessive use of pesticides and fertilizers has led to widespread non-point source pollution [5]. This not only degrades water quality and ecosystem health but also threatens the long-term sustainability of agricultural systems.

In addition to direct toxicity, the overuse of chemical pesticides has also exacerbated the problem of resistance in insect pests and plant pathogens. Repeated pesticide exposure allows resistant pests to survive and reproduce, driving the evolution of various resistance mechanisms and weakening the performance of traditional chemical pesticides [4,6,7]. Molecular diagnostic studies have shown that key pesticide resistance mutations such as G119S and L1014F are now widespread in global pest populations [8,9]. Even in regions practicing organic agriculture, the repeated use of naturally sourced pesticides can impose selection pressure on non-target aquatic organisms, fostering genetic adaptations within those populations [10]. Collectively, these challenges highlight the growing limitations of traditional pesticide-based agriculture and underscore the vital need for innovative and sustainable solutions.

In response to the growing challenges posed by traditional pesticides, researchers increasingly advocate shifting from static and input-driven control strategies to dynamic, nature-based defense systems [11]. The emergence of nanotechnology has brought revolutionary hope for achieving this goal. Nano-pesticides, due to their nanoscale properties, can improve the delivery, stability, and specificity of active ingredients, thereby enhancing efficacy while reducing application rates [1,12]. Despite these advantages, conventional nano-synthesis methods (physical and chemical methods) are energy-intensive and require the use of highly toxic chemicals, which may pose new environmental risks [12].

In this context, green nanotechnology has been gaining immense attention as a sustainable alternative that utilizes biological systems such as plants [13,14,15] and microorganisms to synthesize nanomaterials [16,17]. This method is environmentally friendly and cost-effective, avoiding the introduction of toxic chemical reagents and demonstrating significant ecological compatibility [12,18]. In particular, plant-derived green nano-pesticides have demonstrated excellent insecticidal and fungicidal activities, highlighting their potential in crop protection [14,16]. Additionally, bio-based intelligent nano-delivery systems synthesized from agricultural and forestry waste, such as lignin and cellulose, have demonstrated precise and controlled release of pesticides while simultaneously promoting the valorization of agricultural waste, marking an important step toward closed-loop nanoscale agriculture [19].

Among the various green synthesis systems, microalgae, being unicellular photosynthetic organisms, are emerging as highly efficient “biological factories” for nanomaterial production [3,20]. Microalgae exhibit unparalleled fast growth rates, low cultivation costs, and strong environmental adaptability, and can be mass cultivated in various water bodies, including wastewater [20,21]. More importantly, microalgal cells contain various bioactive molecules such as polysaccharides, proteins, and pigments, which can function as natural reducing and stabilizing agents. These biomolecules can effectively reduce metal ions into nanoparticles while simultaneously preventing their aggregation through inherent capping and stabilization effects [22,23].

Microalgae have been successfully used to synthesize various metal and metal-oxide nanoparticles such as gold (Au), silver (Ag), copper (Cu), zinc (ZnO), iron (Fe_3_O_4_), and palladium (Pd), all of which exhibit notable bioactivity. For instance, AgNPs and AuNPs synthesized by microalgae have demonstrated broad-spectrum antibacterial and antifungal properties [20,24,25,26]. Additionally, nitrogen-fixing cyanobacteria serve not only as a direct natural biofertilizer but can also be combined with nanotechnology to enhance plant nutrition by improving stress resistance and environmental restoration [27,28,29]. Importantly, microalgae-mediated nano-synthesis can be directly combined with wastewater treatment for the simultaneous removal of heavy metals (such as Cr, Pb, and Cd) and degradation of organic pollutants, while converting accumulated metals into valuable nanomaterials, representing an ideal synergy between green synthesis and environmental remediation [3,20,30,31].

Although microalgae-based nanotechnology has immense potential for agricultural applications, current research is still in its infancy and lacks a comprehensive, systematic evaluation. The evaluation of individual microalgae-derived nanomaterials against specific pests has been prioritized in current research, thereby providing only a narrow perspective on their broader agricultural potential [16]. In contrast, there is a lack of discussion on the comprehensive potential of microalgae as a multifunctional nano-biological factory, including their underlying biosynthetic mechanisms, broader roles in pest management and crop health promotion, and their contributions to environmental remediation [12,32].

Therefore, this review provides an integrated and critical overview of microalgae-mediated nanotechnology for sustainable agriculture. We first discuss the biochemical basis of nanoparticle formation in microalgae, focusing on polysaccharides, proteins, photosynthetic pigments, extracellular polymeric substances, and secondary metabolites that participate in metal-ion reduction, nucleation, capping, and stabilization. We then summarize intracellular and extracellular synthesis pathways, advanced synthesis strategies, and key reaction parameters that determine nanoparticle physicochemical properties. Subsequently, we compare major categories of microalgae-derived nanomaterials, including gold, silver, selenium, zinc oxide, bimetallic, and other functional nanoparticles, and evaluate their applications in crop protection, plant growth promotion, and stress mitigation. Particular attention is given to the mechanisms underlying their agronomic advantages, including antimicrobial activity, biofilm disruption, improved nutrient bioavailability, antioxidant regulation, and activation of plant defense responses. Finally, we discuss environmental safety, soil microbial impacts, circular agriculture integration, scale-up challenges, regulatory gaps, and future development directions. By linking microalgal biotechnology, green nanomaterial synthesis, and sustainable agricultural applications, this review aims to clarify the current state of the field and identify key priorities for future research and translation.

## 2. Microalgal Diversity and Biochemical Richness

Microalgae, due to their rich biochemical components and distinct metabolic pathways, are regarded as ideal biological nano-factories [23,33,34]. The process of nanoparticle biosynthesis in microalgae involves a coordinated sequence of reduction, nucleation, and stabilization steps driven by intracellular and extracellular biomolecules. Gaining an in-depth understanding of these mechanisms is essential for controlling nanoparticle properties such as size, morphology, and surface chemistry, as well as optimizing their performance in sustainable agriculture applications such as nano-pesticides and nano-fertilizers [35,36,37].

### 2.1. Polysaccharides

Polysaccharides play a crucial dual role during microalgae-mediated nanoparticle synthesis. Due to their abundant functional groups, such as hydroxyl and aldehyde groups, microalgal polysaccharides can effectively reduce metal ions to the atomic state by donating electrons [38,39,40,41]. Simultaneously, polysaccharides can rapidly adsorb onto the surface of newly formed nanoparticles, forming a coating layer, which effectively prevents particle agglomeration through the spatial steric hindrance effect, thereby ensuring colloidal stability [36,42,43]. For example, soluble polysaccharides extracted from *Chlorella vulgaris* were successfully used to synthesize and stabilize AgNPs [44]. Different microalgal polysaccharides, owing to variation in their molecular composition, functional group density, and molecular weight, can mediate the synthesis of diverse nanoparticles and strongly influence their size, morphology, and stability. In general, sulfated polysaccharides have been shown to often exhibit strong metal-binding and reducing capabilities, while cyanobacterial cell-wall polysaccharides display exceptionally high affinities for metal adsorption and reduction for the production of various nanoparticles when compared to other microorganisms [23,45,46,47].

### 2.2. Proteins

Apart from polysaccharides, proteins and enzymes also play an indispensable role in nanoparticle synthesis. Protein molecules are made up of amino acid residues that contain hydroxyl, carboxyl, and amine groups, which chelate metal ions, allow electron transfer, and promote metal ion reduction [25,48]. Enzymatic activities can further elevate this process, particularly through redox-active proteins involved in cellular metabolism. For example, oxidoreductases such as ATP synthase and superoxide dismutase have been observed to directly participate in the biological synthesis and stabilization of AgNPs in *Chlamydomonas reinhardtii* [49,50].

Studies have shown that protein-depleted algal extracts consistently show altered synthesis rates and particle size and size distribution of nanoparticles, indicating that intracellular proteins are essential regulators in the biological synthesis process of NPs [49]. Notably, even extremely low concentrations of specific protein fractions have been reported to efficiently drive nanoparticle formation, as illustrated by the synthesis of ultrasmall CuO nanoparticles (2 nm) from protein components isolated from *Macrocystis pyrifera* with extremely low overall protein content (0.72 μg) [51].

### 2.3. Pigments

Photosynthetic pigments also play an active and mechanistically important role in microalgae-mediated nanoparticle biosynthesis. Pigment-protein complexes such as phycocyanin, phycobilins, chlorophylls, and carotenoids possess strong redox potential and can participate directly in metal-ion reduction, particularly under light irradiation. Phycocyanin and C-phycobilin from cyanobacteria can directly reduce metal salts to form AgNPs and CdS nanoparticles [23,52,53]. The involvement of photosynthetic pigments is closely linked to light-driven electron transfer processes. Light energy can excite these pigment molecules, causing them to generate high-energy electrons, which are then used to reduce metal ions [23,54]. In green algal cells, pigments such as chlorophyll *a* and β-carotene can not only serve as a direct electron source themselves but can also transfer electrons through molecules such as NADH, thereby enhancing enzymatic reduction pathways and enabling the efficient synthesis of nanoparticles such as AuNPs [55]. Nanoparticle formation is accompanied by a marked decrease in chlorophyll content and fluorescence intensity, indicating the consumption of photosynthetically generated electrons during the reduction process [56]. Collectively, these findings underscore the role of photosynthetic pigments as dynamic electron sources rather than passive cellular components.

### 2.4. Extracellular Polymeric Substances (EPS)

Extracellular polymeric substances (EPS) are critical mediators of microalgae-derived nanoparticle synthesis and stabilization. Carbohydrate-rich EPS from microalgae such as *Graesiella emersonii* and *Chlorella* sp. can act simultaneously as reducing and capping agents, enabling one-step formation of stable silver and gold nanoparticles [57,58,59]. The presence of EPS is essential for maintaining colloidal stability, as its removal leads to rapid nanoparticle aggregation, whereas EPS-containing systems yield particles with controlled size and morphology [60,61]. Moreover, EPS composition strongly influences nanoparticle quality; protein-enriched EPS from *Arthrospira platensis* produces AgNPs with improved monodispersity and more uniform surface passivation, underscoring the pivotal role of EPS chemistry in microalgae-mediated nano-synthesis [62].

### 2.5. Secondary Metabolites

Various other metabolites have been successfully shown to participate in the reduction, stabilization, and termination stages of nano-synthesis. For instance, various fatty acid esters (e.g., methyl palmitate and methyl oleate) and hydrocarbons were detected from the extract of the novel microalgae *Coelastrellia aeroterrestrica*, which have been proposed to act primarily as terminating agents during the biosynthesis of AgNPs [63]. Other studies on microalgae (e.g., *Isochrysis* sp. and *Desmodesmus abundans*) have also clearly demonstrated synergistic involvement of fatty acid esters, together with proteins, nucleic acids, amino acids, and carbohydrates, in enhancing the biological reduction and stabilization of silver nanoparticles, with lipid-derived compounds contributing to surface capping and termination, while nitrogen- and oxygen-containing biomolecules provide the primary reducing functionality [64,65].

As summarized in Table 1, most biomolecules found in microalgae, such as polysaccharides, proteins, and pigments, through their abundant functional groups (-OH, -COOH, and -NH_2_), can either work individually or in synergy for the synthesis of nanoparticles. These groups mediate the reduction in metal ions to their elemental forms and subsequently serve as natural end-capping agents, coating the nanoparticle surface to enhance colloidal stability and prevent agglomeration [25,33,66]. Among these components, carotenoids, due to their excellent electron donor properties, successfully synthesized multi-dispersed spherical and triangular AuNPs within 24 h. Protein extracts also display notable reducing potential, although their activity appears temperature-dependent, suggesting the involvement of enzyme-mediated processes. In contrast, polysaccharide extracts demonstrated species-specific behavior in which cyanobacterial polysaccharides could effectively reduce gold ions, while the polysaccharide from green algae could not do so [67,68]. Thus, differences between algal species also affect how nanoparticles are formed, making it possible to control and tailor nanoparticle properties through the targeted selection of algal species or specific cellular fractions.

## 3. Green Synthesis Pathways for Microalgae-Mediated Nanomaterials

### 3.1. Intracellular vs. Extracellular Synthesis Pathways

According to the location of nanoparticle formation, microalgae-mediated synthesis occurs via two fundamentally distinct pathways, namely intracellular and extracellular synthesis [34,74,75]. Intracellular synthesis occurs within living algal cells. This process begins with the passive adsorption or active transport of metal ions into the cells [76,77]. Once inside the cells, the metal ions are reduced to atoms by inherent reducing agents produced during the microalgal cellular metabolic process (e.g., NADPH generated during photosynthesis) [78,79]. Subsequently, these atoms undergo nucleation and growth, eventually forming nanoparticles within the cells. Nanoparticle formation is spatially regulated within the cell and closely linked to metabolically active regions. For example, transmission electron microscopy and fluorescence-based analyses have revealed that metallic nanoparticles predominantly localize near the cell membrane, organelle-rich regions, or intracellular storage sites of microalgae cells, suggesting the involvement of membrane-associated enzymes, redox-active pigments, and intracellular electron transport processes [80]. Moreover, changes in pigment composition and the preferential formation of nanoparticles near sites for ATP synthesis and energy-intensive cellular zones (e.g., flagella) further support the role of oxidoreductases and photosynthetically derived reducing equivalents in driving intracellular metal reduction [49,56]. However, the primary limitation of intracellular synthesis lies in downstream processing challenges. Efficient recovery of intracellular nanoparticles typically requires cell-disruption techniques, such as mechanical or chemical treatments, which increase process complexity and cost and may also promote nanoparticle aggregation or contamination [34,81].

Extracellular synthesis mainly relies on metabolites secreted by microalgae into the surrounding environment or based on the active groups on the cell wall surface to mediate metal ion reduction and nanoparticle stabilization [76,82]. This process can be carried out in two ways: using cell-free filtrates or complete algal biomass. In cell-free systems, the filtrates, which are rich in extracellular polymeric substances (EPS) or extracted biochemical molecules, including polysaccharides, proteins, and enzymes, directly interact with the metal precursor solutions to reduce metal ions and simultaneously stabilize the resulting nanoparticles [83]. When using whole algal biomass, the negatively charged cell wall (rich in functional groups such as hydroxyl and carboxyl) first adsorbs positively charged metal ions through electrostatic interaction. Subsequently, enzymes or other related biomolecules are employed on the cell wall for reduction, forming nanoparticles that may adhere to the cell surface or be released into the culture medium [84]. Different extracts and variations in extraction methods, culture conditions, and biomass pretreatment have been shown to significantly influence nanoparticle size, stability, and dispersity, highlighting the importance of biochemical composition and processing strategies. For example, the boiled extract of *Desmodesmus* sp. was found to synthesize smaller (3–6 nm) and more stable nanoparticles of AgNPs when compared to the crude extract [80]. Notably, the successful use of extracts from defatted *Nannochloropsis oculata* residual biomass for synthesizing AgNPs and nano-zero-valent iron (nZVIO) underscores the potential of extracellular synthesis for integrating nanoparticle production into biorefinery frameworks [85].

**Figure 1:**
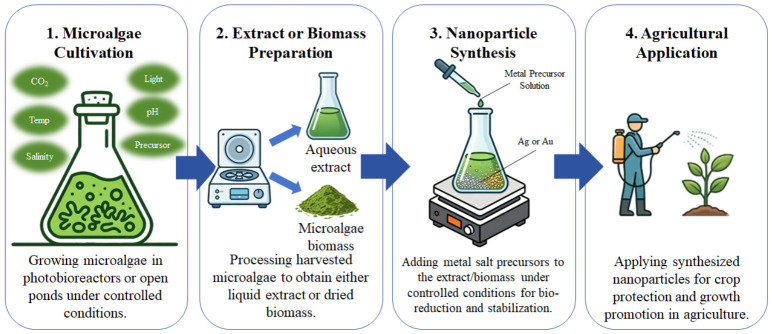
Schematic diagram illustrating the key stages involved in microalgae-mediated green synthesis of nanoparticles.

In addition, further comparative studies of intracellular and extracellular synthesis pathways indicate clear trade-offs between reaction kinetics and process practicality. Extracellular synthesis has been reported to be much slower (7–10 days) than intracellular methods but does offer significant advantages in downstream processing due to simplified recovery and purification steps [67]. In addition, nanoparticle synthesis efficiency and the preference for intracellular or extracellular pathways are strongly governed by culture physiological conditions. Variations in factors such as CO_2_ availability can markedly alter metabolic fluxes and biomolecule distribution, thereby shifting the dominant synthesis route. Overall, extracellular synthesis is preferred due to its operational simplicity, no need for cell disruption, ease of downstream purification, and large-scale production, thereby offering greater translational potential for sustainable nanomaterial production [34,86]. Figure 1 provides a detailed description of the environmental factors affecting microalgal growth and the pathway of microalgae-based extracellular synthesis of nanoparticles.

### 3.2. Advanced Strategies for Microalgae-Mediated Nanomaterial Synthesis and Characterization

To produce nanomaterials with specific sizes, morphologies, and functional properties to meet the diverse demands in sustainable agriculture for promoting plant growth, disease prevention, and alleviating adverse environmental stresses, researchers have developed various synthesis strategies. Precise control over cultivation and reaction conditions, coupled with systematic physicochemical and biological characterization, enables a deeper understanding of nanoparticle formation mechanisms and facilitates the rational optimization of their performance in sustainable agricultural applications [23,87,88].

#### 3.2.1. Aqueous Extract/Supernatant-Mediated Synthesis

Synthesis mediated by standard aqueous extracts or cell-free supernatants represents the most fundamental and widely adopted method for the synthesis of nanomaterials by microalgae (Figure 1). This method usually involves harvesting microalgae biomass followed by drying and grinding, after which aqueous extracts are produced through disruptive methods such as heating or ultrasonic-assisted extraction. Alternatively, cell-free supernatants can be directly collected from culture media. In both cases, the resulting solutions are rich in bioactive molecules such as proteins, polysaccharides, polyphenols, pigments, and other metabolites that can work together as reducing agents and stabilizers to convert metal ions into nanoparticles and maintain their colloidal stability. Multiple studies have reported that biomolecules can effectively mediate the synthesis of a wide range of metal and metal oxide nanoparticles, including gold, silver, iron oxide, zinc oxide, and magnesium oxide [89,90]. The resulting nanoparticles often exhibit controlled size distributions, diverse morphologies, and high dispersity, and they frequently display enhanced functional properties, such as catalytic, antimicrobial, or photocatalytic activities.

Cyanobacteria (blue-green algae) also represent valuable biological resources for green nanoparticle synthesis. For example, silver nanoparticles synthesized from the extract of *Spirulina platensis* have been shown to exhibit physicochemical characteristics and antibacterial activity comparable to those of chemically synthesized counterparts, while offering superior stability and biocompatibility [91]. By regulating the pH value, gold nanoparticles of different morphologies (spherical, triangular, hexagonal) and even nanorods can be synthesized using blue-green algae [92]. Overall, aqueous extract- or supernatant-mediated synthesis is a simple and mild approach; however, its efficiency is significantly affected by factors such as the algal species, extraction conditions, and pH, temperature, and reactant concentration in the reaction system [57,87,93].

#### 3.2.2. Gamma-Ray Assisted Synthesis

Gamma-ray-assisted synthesis is an advanced green strategy that utilizes high-energy radiation to trigger reduction reactions. Compared with the standard wet chemical method, gamma radiation can uniformly generate reducing species (such as hydrated electrons) at normal temperature and pressure, enabling a rapid and controllable reduction process without the need for additional reducing agents or high-temperature conditions. Studies have shown that using polysaccharides extracted from *Spirulina platensis* as a stabilizing agent, silver nanoparticles with an average particle size of 25.25 nm, spherical shape, and good dispersion were successfully prepared under 10.0 kGy of gamma radiation [94]. This method not only improves the synthesis efficiency but also effectively regulates the particle size distribution of nanoparticles by controlling the radiation dose, providing a green and efficient approach for large-scale production of uniform and stable nanomaterials.

#### 3.2.3. Solid-Phase Synthesis

For industrial applications, solid-phase synthesis has attracted increasing attention due to its solvent-free operation, high efficiency, and environmental compatibility. This method synthesizes nanoparticles by mechanically grinding and mixing microalgae powder with metal precursors and activators (such as NaOH) together to enable direct solid–solid reactions that lead to nanoparticle formation. Studies have confirmed that by grinding *Spirulina* powder with silver nitrate solid in the presence of NaOH, silver nanoparticles with a particle size of approximately 19.9 nm and good dispersion can be synthesized in one step [95]. The proteins in *Spirulina* (such as the hydroxyl groups of amino acids) simultaneously act as reducing agents and stabilizers during this process. This technology significantly simplifies the production process, reduces costs, and avoids secondary wastewater generation, demonstrating extremely high potential for industrial large-scale production.

**Figure 2:**
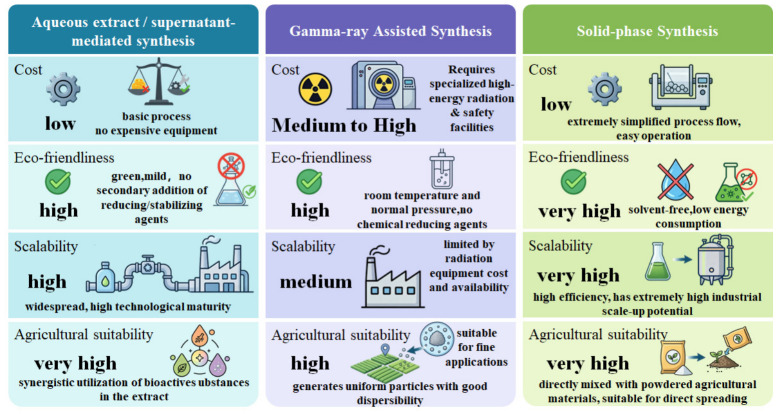
Comparative analysis of three nanomaterial green synthesis technologies.

Figure 2 outlines all three green synthesis routes for microalgae-mediated nanoparticle production discussed above, including aqueous extract-based bio-reduction, gamma-ray-assisted radiolytic reduction, and solvent-free solid-state synthesis. These approaches utilize microalgal biomass in different forms and enable tunable control over nanoparticle size, morphology, and stability.

### 3.3. Influence of Reaction Conditions on Nano-Synthesis

#### 3.3.1. Temperature

Temperature has also been shown to significantly affect the growth and metabolism of microalgae. Temperature stress can shift cellular resource allocation, often reducing overall biomass productivity while promoting the accumulation of specific metabolites, such as lipids. A study on *Nannochloropsis oculata* showed that heat stress (35 °C) reduced biomass productivity but enhanced lipid accumulation compared with the optimal temperature (25 °C). Notably, the lipid-extracted residual biomass was subsequently used to synthesize AgNPs and nano-zero-valent iron (nZVI), which exhibited high efficiency in removing heavy metals (Pb^2+^ and Cd^2+^) [85]. These results indicate that temperature-induced metabolic shifts can improve the functional value of post-extraction algal residues for nanomaterial synthesis and environmental remediation.

Apart from cell growth and metabolism, temperature also plays a significant role in reaction kinetics during nanoparticle synthesis by regulating metal-ion reduction rates, nucleation frequency, and particle growth. In general, an increase in temperature accelerates the reduction in metal ions and increases the nucleation rate, which results in the formation of smaller nanoparticles at higher yield [96,97]. However, temperature also plays a critical role in determining nanoparticle morphology and stability, as excessive thermal energy can alter growth pathways or promote particle aggregation [57]. Furthermore, excessively high temperatures have also been shown to denature or decrease the activity of biological reducing agents (such as enzymes) that can disrupt capping interactions or induce agglomeration of nanoparticles [75,98].

#### 3.3.2. pH

Solution pH is also a key factor influencing the size and morphology of nanoparticles. Generally, an alkaline environment is conducive to the formation of monodisperse, small-sized nanoparticles, while an acidic environment tends to promote slower reduction kinetics and produce anisotropic, larger-sized particles [75,99]. For example, in the study using *Haematococcus pluvialis* CFS to synthesize AgNPs, it was found that pH 11 was the most effective synthesis condition, and a strong reaction could be triggered within 15 min, while the reaction under acidic conditions was inhibited [57]. This is because a high pH value is beneficial for the deprotonation of functional groups on the cell surface and biological molecules, thereby enhancing their electrostatic adsorption and reduction ability for metal ions [3]. Studies have shown that when the pH value increases from 4 to 10, the size distribution of AgNPs synthesized by *Parachlorella kessleri* narrows from 20 to 60 nm to around 15 nm [100]. Spectroscopic analyses further indicate that pH not only controls reaction efficiency but also directly influences nanoparticle optical responses, reflecting changes in particle size and aggregation state [33,46,65].

#### 3.3.3. Metal Precursors

Within an optimal range, increasing precursor concentration enhances nanoparticle yield; however, excessive concentrations can promote particle growth, broaden size distribution, and induce agglomeration due to insufficient capping capacity [34,101]. In whole-cell systems, both precursor concentration and the ratio of algal biomass to metal ions critically influence synthesis efficiency and product stability, as cellular adsorption and reduction capacities must be balanced against metal ion loading [102]. Therefore, in order to achieve efficient and controllable synthesis, it is necessary to optimize the ratio of microalgae biomass to the precursor salt [103].

## 4. Diversity of Microalgae-Derived Nanomaterials for Agriculture

In the context of sustainable agricultural applications, microalgae-derived nanomaterials encompass a diverse range of compositions, including gold, silver, selenium, zinc oxide, and various composite nanostructures. The physicochemical properties and application potential of these nanomaterials vary widely depending on the core metal composition, the microalgal species employed, and the specific synthesis conditions. This inherent biological and process-level tunability offers significant opportunities to design nanomaterials with tailored characteristics for targeted agricultural functions [104].

### 4.1. Gold Nanoparticles

The biological synthesis of gold nanoparticles (AuNPs) is primarily driven by the reduction of Au^3+^ ions by redox-active biomolecules present in microalgal extracts, including proteins, polysaccharides, pigments, and associated metabolites [105]. These molecules donate electrons to gold ions, initiating nanoparticle nucleation, while simultaneously adsorbing onto the nascent particle surface to provide stabilization. AuNPs have demonstrated significant potential for various applications in the agricultural field due to their unique physical and chemical properties, good biocompatibility, and low toxicity. The benefits include promoting crop growth, providing antioxidant defense, and exhibiting antifungal activity [106,107]. Studies have shown that wheat treated with AuNPs derived from cyanobacteria *(Spirulina platensis*) can significantly increase its yield and nutritional value [107]. Additionally, bio-stabilized colloidal AuNPs produced by *Chlorella* species have been reported to enhance plant antioxidant capacity and catalytic activity. AuNPs synthesized from red algae (*Gelidiella acerosa*) have shown significant metabolic enzyme inhibition and antioxidant activity, indicating their ability to regulate redox-related pathways [108]. These effects are thought to be associated with the interaction of AuNPs with photosynthetic pigments, including chlorophyll, which may facilitate improved redox balance and metabolic efficiency in plants [109]. Also, these findings indicate that AuNPs can directly inhibit pathogens as well as enhance the plant’s own resistance by regulating the plant’s antioxidant system.

### 4.2. Silver Nanoparticles

Silver nanoparticles (AgNPs) are extensively studied and technically mature nanomaterials produced by microalgae. Their primary value in the agricultural field lies in their broad-spectrum and highly efficient antibacterial activity against plant pathogens [20,22]. AgNPs synthesized by microalgae typically exhibit high colloidal stability and enhanced biocompatibility due to natural biomolecular capping, which reduces phytotoxicity and environmental risk [110]. Numerous studies have demonstrated that AgNPs synthesized from different microalgal and cyanobacterial species effectively inhibit both Gram-positive and Gram-negative pathogens, often showing superior performance compared with conventional antibiotics or silver salts [106,111,112,113]. Beyond in vitro antibacterial efficacy, microalgae-based AgNPs have also been successfully applied in agricultural contexts, such as seed coating and seed pre-treatment, where they significantly suppress soil-borne and seed-borne diseases while, in some cases, simultaneously promoting early plant growth [113,114]. The antibacterial effectiveness of these AgNPs is attributed to their small size, high surface reactivity, and biomolecule-derived surface functionalization, which together enhance their interaction with microbial cells. Microscopic analyses indicate that AgNPs disrupt bacterial cell wall integrity, induce membrane damage, and cause leakage of intracellular contents, ultimately leading to cell death [94]. In addition, the natural biomolecular capping provided by microalgae not only improves nanoparticle stability and dispersibility but also contributes to enhanced antimicrobial potency at relatively low dosages.

### 4.3. Selenium Nanoparticles

Selenium (Se) is an essential trace element for both humans and animals, and selenium nanoparticles (SeNPs) have attracted increasing attention in agricultural research as innovative bio-stimulants and plant protection agents, owing to their relatively low toxicity, high bioavailability, and enhanced biological activity compared with conventional selenium salts [115,116]. Recent studies have also shown that SeNPs can serve as effective antioxidants and fertilizer additives, promoting plant growth and alleviating oxidative stress [117]. Foliar spraying of SeNPs has been shown to significantly enhance crop yield and nutritional value. Long-term field trials have demonstrated that spraying SeNPs at different growth stages of wheat not only significantly increased wheat yield and the selenium content in seeds (up to 32 times) but also improved the contents of starch and soluble sugars in seeds [118]. Beyond selenium biofortification, SeNPs have been reported to improve physiological performance and fruit quality parameters in horticultural crops even under non-stress conditions, indicating their role as growth-promoting bio-stimulants [119,120]. In addition, SeNPs synthesized from the extract of *Spirulina platensis* (SP-SeNPs) also consistently displayed strong antioxidant properties, including free-radical scavenging activity against multiple reactive oxygen species [121,122]. These properties endow SeNPs with great potential for alleviating both biotic and abiotic stresses in plants. Foliar application of SeNPs has been shown to strengthen antioxidant defense systems of plants by reducing oxidative stress markers while increasing photosynthetic pigments, phenolic compounds, and free-radical scavenging capacity, thereby alleviating damage caused by salinity [123]. These benefits are largely attributed to selenium’s role as a functional component of antioxidant enzymes, which enhances reactive oxygen species (ROS) detoxification and protects cellular integrity [124].

### 4.4. Zinc Oxide and Manganese-Zinc Dual-Metal Nanomaterials

Zinc oxide nanoparticles (ZnONPs) synthesized using microalgal biomass (e.g., Chlorella) benefit from intrinsic biomolecules such as proteins and polysaccharides that can effectively stabilize and improve their biological compatibility [125]. The biosynthesized ZnONPs from various algal and cyanobacterial sources can be tailored to different shapes and sizes and are found to consistently exhibit strong antimicrobial and antioxidant activities, underscoring their multifunctional role in plant protection and stress mitigation [125,126]. Biologically synthesized ZnONPs exhibit strong antimicrobial activity, supporting their relevance for both agricultural and agri-food applications [127]. In crop systems, low-dose ZnONP treatments have been shown to significantly improve seed germination and seedling vigor, likely by enhancing zinc bioavailability and stimulating early metabolic activity. However, elevated concentrations can induce phytotoxic effects, underscoring a narrow optimal dosage window [128].

Bimetallic nanomaterials typically exhibit superior synergistic effects compared to single-metal nanomaterials. A recent innovative study successfully synthesized zinc oxide-manganese bimetallic nanoparticles (ZnO-MnNPs) using a mixed extract of *Spirulina* and *Chlorella* sp., demonstrating excellent colloidal stability and monodispersity [129]. Preliminary in vitro antifungal tests indicate that ZnO-MnNPs have measurable inhibitory activity against tomato pathogenic fungi such as *Sclerotinia sclerotiorum* and *Fusarium equiseti*. Although the effect is relatively mild, their high stability and biocompatibility suggest their potential as sustainable antifungal agents in agricultural practices [129]. In parallel, other algae-mediated bimetallic systems, such as Cu–Fe nano-composites synthesized using *Chlorella*, have shown effectiveness in photocatalytic pollutant degradation, underscoring the broader potential of microalgae-derived bimetallic nanomaterials in both crop protection and environmental remediation [130].

### 4.5. Other Nanomaterials

In addition to the major classes of nanomaterials discussed above, microalgae can also be employed to synthesize a range of other functional nanomaterials with emerging agricultural applications. Copper-based nanoparticles (CuONPs) biosynthesized using *Chlamydomonas* sp. have been proven to possess antibacterial and antiviral activities, highlighting their potential for crop disease management [131]. Similarly, algal systems, including *Chlorella vulgaris*, have been used to produce AgCl nanoparticles with strong antimicrobial properties [132]. Iron-based (α-Fe_2_O_3_) nanoparticles represent another important category. Biogenic α-Fe_2_O_3_ nanoparticles have been shown to enhance seed germination, early seedling growth, and photosynthetic performance by modulating reactive oxygen species and chlorophyll synthesis in cereal crops such as rice and maize [133]. Under field conditions, spraying low concentrations (10 mg/L) of α-Fe_2_O_3_ nanoparticles on the leaf surface significantly increased the chlorophyll content and yield of rice and corn [133]. In addition, magnetic iron oxide nanoparticles (Fe_3_O_4_) synthesized from brown algae, including *Sargassum muticum* and *Padina pavonica*, have demonstrated effectiveness in heavy-metal adsorption and show potential as carriers for targeted delivery systems [47,134]. In addition to metal nanoparticles, microalgae and cyanobacteria have been employed to produce semiconductor nanomaterials such as cadmium sulfide (CdS) quantum dots. CdS quantum dots biosynthesized using hemoglobin-like proteins from cyanobacteria (*Phormidium tenue*) exhibit strong fluorescence properties, enabling their use as bioimaging probes and sensors. These optically active nanomaterials show promise for applications in plant cell imaging, pathogen detection, and biosensing, thereby extending the utility of microalgae-mediated nanomaterials beyond crop protection into agricultural diagnostics and monitoring [53].

## 5. Applications in Crop Protection and Growth Promotion

**Figure 3:**
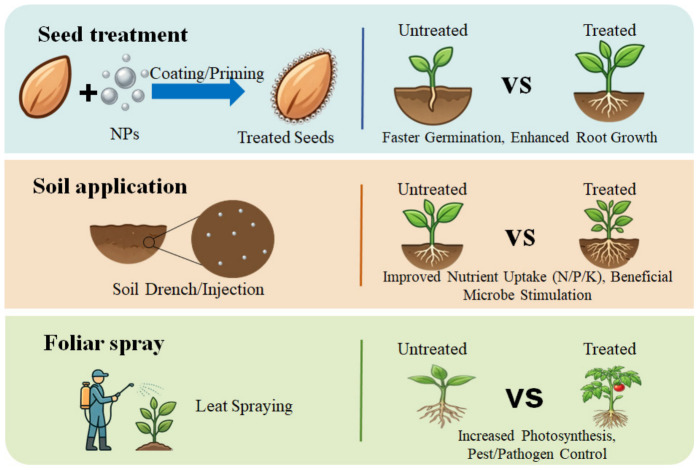
Three major agricultural delivery routes of nanoparticles and their agronomic advantages.

Nanoparticles synthesized by microalgae have shown great potential in sustainable agriculture. They can not only be used as green pesticides to prevent plant diseases but also as nano-fertilizers and biological stimulants to promote crop growth and enhance the crop’s tolerance to non-biological stress (Figure 3). Their mechanisms of action are diverse, encompassing antimicrobial activity, improved nutrient-use efficiency, and modulation of plant physiological and antioxidant responses that allow for a broad spectrum of applications in agriculture.

### 5.1. Major Agronomic Advantages

#### 5.1.1. Control of Bacterial Diseases

**Figure 4:**
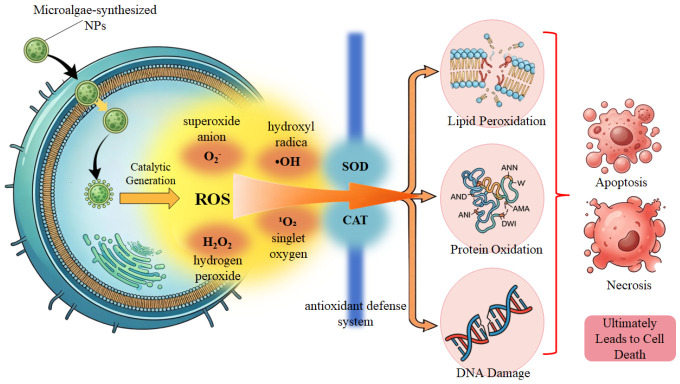
Proposed antibacterial mechanism of green-synthesized nanoparticles against Gram-negative bacteria.

Microalgae-derived nanoparticles exhibit significant inhibitory effects on various plant pathogenic bacteria, highlighting their potential for crop disease management. Notably, green-synthesized silver nanoparticles exhibit high antimicrobial potency at low dosages, outperforming both metal salts and algal extracts alone [94]. Scanning electron microscopy (SEM) analysis indicates that these nanoparticles interact directly with bacterial cell surfaces, disrupting membrane integrity and inducing cellular leakage, which ultimately leads to pathogen inactivation [94]. In addition, antibacterial effectiveness is strongly size-dependent, with smaller nanoparticles exhibiting greater inhibitory activity against both Gram-positive and Gram-negative phytopathogens [135]. Figure 4 illustrates the antibacterial mechanism of green-synthesized AgNPs, showing their attachment to Gram-negative bacterial membranes, intracellular penetration, and induction of reactive oxygen species, which cause oxidative damage to cellular components and ultimately lead to bacterial cell death.

#### 5.1.2. Control of Fungal Diseases

Microalgae-mediated nanoparticles have shown considerable potential for suppressing major fungal pathogens responsible for crop diseases. These nanomaterials act through a combination of direct antifungal activity and induction of host plant defense responses, offering a sustainable alternative to conventional fungicides. In the case of *Fusarium* wilt, one of the most destructive soil-borne diseases affecting tomato, nanoparticle-based treatments have demonstrated strong protective effects [136]. Apart from organic nanoparticles, microalgae-derived metal and metalloid nanoparticles have also exhibited antifungal efficacy. Iron oxide (Fe_3_O_4_) nanoparticles synthesized by *Chlorella* K01 species showed significant inhibitory activity against various *Fusarium* species [137]. Similarly, selenium nanoparticles (SeNPs) extracted from the extract of the cyanobacteria *Desmonostoc alborizicum* demonstrated antifungal activity against various fungi, with *Fusarium oxysporum* among the most sensitive species tested [138].

In addition, the combination of microalgal extracts with complementary functional materials offers an effective strategy to enhance antifungal performance through synergistic interactions. For example, a chitosan–magnesium–algal nano-composite synthesized using *Ulva fasciata* extract, chitosan, and magnesium nanoparticles exhibited markedly higher in vitro antifungal activity against *Macrophomina phaseolina* than either the algal extract or magnesium nanoparticles alone, accompanied by pronounced morphological deformation of fungal hyphae [139]. Pot experiments further demonstrated that seed treatment with this nano-composite significantly reduced bean rot severity across multiple growing seasons, confirming its efficacy under near-field conditions [139].

#### 5.1.3. Control of Viral Disease

Microalgae-mediated nanoparticles have also shown emerging potential in the management of plant viral diseases. Gold nanoparticles synthesized using algal systems have demonstrated antiviral activity in model studies, indicating their ability to interfere with viral infection processes [131]. In agricultural contexts, both silver and gold nanoparticles derived from brown algal polysaccharides have exhibited significant inhibitory effects against tobacco mosaic virus (TMV) under in vitro conditions [140].

Table 2 provides a comparative overview of the antibacterial activity of microalgae-derived nanoparticles against major agricultural pathogens, summarizing nanoparticle types, target bacteria, and key antimicrobial mechanisms, including ROS generation, membrane disruption, and metabolic interference.

### 5.2. Mechanisms Underlying Agronomic Advantages

#### 5.2.1. Disruption of Biofilm Formation

Biofilm formation is a critical virulence strategy employed by many plant pathogenic bacteria, contributing to enhanced survival, persistence on plant surfaces, and resistance to conventional antimicrobial treatments. Nanoparticles derived from microalgae can effectively destroy biofilms, offering an effective approach to suppress pathogen virulence rather than solely targeting cells directly. For instance, ZnONPs synthesized from the cyanobacteria *Nostoc* sp. EA03 have demonstrated anti-biofilm activity against *Escherichia coli*, *Staphylococcus aureus*, and *Pseudomonas aeruginosa* [144]. These nanoparticles are believed to interfere with extracellular polymeric substances, impair cell–cell adhesion, and damage microbial cell membranes, thereby reducing biofilm integrity and pathogenic potential.

#### 5.2.2. Plant Growth Promotion and Yield Enhancement

Microalgae-derived nanoparticles exhibit considerable potential as nano-fertilizers and bio-stimulants that enhance early plant development and overall growth performance. Numerous studies demonstrate that nanoparticle treatments derived from microalgae can significantly improve seed germination, seedling vigor, and photosynthetic capacity across a range of crop species. Iron- and zinc-based nanoparticles, in particular, have been shown to stimulate germination, root and shoot elongation, chlorophyll accumulation, and biomass production when applied at low and optimized concentrations [135,143,145,146]. These effects are attributed to improved micronutrient bioavailability, enhanced chlorophyll biosynthesis, and stimulation of key physiological and metabolic processes during early growth stages.

On the other hand, silver-and magnesium-based nanoparticles synthesized have shown growth-promoting properties, including increased plant height, root development, leaf expansion, and yield-related traits. For example, treating wheat and bean seeds with AgNPs synthesized from the soluble polysaccharides of *Chlorella vulgaris* increased plant height, root length, and leaf area by approximately 23%, 30%, and 60%, respectively [44]. Field-based studies have demonstrated that microalgae-derived nano-fertilizers can significantly enhance crop productivity and nutritional quality. For example, a nano-iron biological fertilizer formulated from spindle-shaped iron nanoparticles biosynthesized using *Spirulina* biomass markedly increased rice yield and grain iron content, outperforming conventional N/P/K fertilization strategies [147]. Similarly, foliar application of *Scenedesmus obliquus* extracts and their corresponding ZnO nanoparticles substantially improved tomato fruit yield and quality attributes [148].

#### 5.2.3. Abiotic Stress Mitigation

The nanoparticles derived from microalgae can also improve crop resilience to increasingly severe abiotic stresses. Under high saline conditions, treatments based on *Arthrospira* have been shown to enhance plant salt tolerance by promoting the accumulation of compatible solutes, maintaining ionic homeostasis (K^+^/Na^+^ balance), strengthening antioxidant capacity through increased reactive oxygen species (ROS) scavenging, and upregulating genes associated with photosynthesis and hormone signaling. These coordinated physiological and molecular responses collectively improve seed germination and seedling establishment under salt stress [149]. Similarly, foliar application of silver nanoparticles biosynthesized from *Spirulina* extracts has been reported to alleviate oxidative damage and stimulate growth in salt-stressed pearl millet seedlings [150]. In addition, the application of nanomaterials such as SiNPs and ZnONPs can activate the antioxidant defense system of crops and accumulate osmotic regulatory substances, thereby enhancing their tolerance to drought stress [3]. Similarly, Fe_3_O_4_NPs and SiO_2_NPs have also been found to help improve the tolerance of crops to heavy metal (such as cadmium and lead) stress by reducing heavy metal absorption, promoting chelation, and compartmentalization to alleviate their toxic effects [3].

#### 5.2.4. Induction of Plant Immunity

Beyond their direct effects of agronomic advantages, microalgae-derived nanoparticles can also act as inducers to activate the plant’s own immune defense system, namely induced systemic resistance (ISR) and systemic acquired resistance (SAR) [37,151]. This mechanism involves the activation of a series of signaling pathways within the plant that ultimately leads to the upregulation of key defense genes. The salicylic acid (SA) pathway is the key mediator of SAR, which induces the expression of pathogenesis-related (PR) genes and enhances resistance to biotrophic pathogens [37]. In contrast, ISR is mainly associated with the jasmonic acid (JA) and ethylene (ET) signaling pathways, which are commonly activated during defense against necrotrophic pathogens and herbivorous insects [37].

Treatment with microalgae-based nanoparticles has been shown to significantly enhance the expression of defense-related genes in various crops, such as tomatoes. For instance, treatment with Ag/chitosan nano-complex (Ag/CHI NCsw) was also seen to enhance the expression of several defense-related genes in tomatoes, such as *chitinase*, *glutathione-S-transferase* (*GST*), *PAL1*, and *defensin*, thereby enhancing the resistance to the pathogen *Rhizoctonia solani* [151]. Additionally, AuNPs synthesized by *Cladosporium cladosporioides* have also been proven to activate the plant’s defense response, further supporting the role of biologically derived nanoparticles as immune-priming agents that strengthen plant resistance through transcriptional regulation of defense pathways [29]. The proteins encoded by these activated defense genes have multiple functions: PR1 is a key marker of SAR [151]; chitinase can hydrolyze the main component of fungal cell walls, chitin [29,151]; and PAL is a key enzyme in the phenylpropanoid metabolic pathway and is involved in the synthesis of antibacterial compounds such as lignin, coumarin, and phytoalexin, while LOX is involved in the synthesis of jasmonic acid [15].

**Figure 5:**
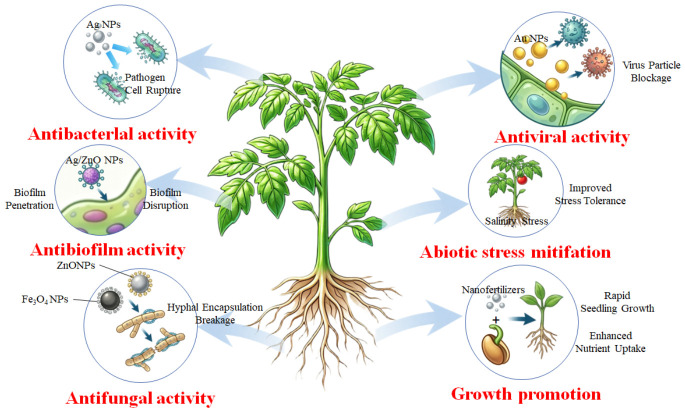
Multifunctional Roles of Microalgae-Mediated Nanotechnology in Sustainable Agriculture.

Figure 5 summarizes the multifunctional roles of microalgae-derived nanoparticles in sustainable agriculture, highlighting their antibacterial, antifungal, antiviral, and antibiofilm activities, as well as their function as nano-fertilizers and bio-stimulants that enhance nutrient uptake, stress tolerance, and overall crop productivity.

## 6. Environmental Impact, Safety, and Circular Economy

As microalgae-mediated nanotechnology continues to show expanding potential in agricultural applications, increasing attention must be directed toward its environmental fate, biosafety, and life-cycle sustainability. Key considerations include the behavior and persistence of these nanomaterials in soil and plant systems, their potential impacts on non-target organisms and beneficial microorganisms, and their long-term ecological consequences.

### 6.1. Plant Toxicity and Biocompatibility

Plant toxicity and biocompatibility are critical determinants governing the safe and effective application of nanomaterials in agricultural systems. Compared with traditional chemical synthesis methods, green-synthesized nanoparticles, especially those synthesized by microalgae, typically exhibit lower toxicity and higher biocompatibility due to the presence of active molecules such as proteins, polysaccharides, and other organic compounds on their surfaces. The core advantage of green synthesis lies in the elimination of toxic chemical reagents commonly used in conventional fabrication processes. Traditional chemical synthesis methods often involve toxic reducing agents and stabilizers such as sodium borohydride (NaBH_4_), which are intrinsically toxic and whose residual by-products can significantly increase the environmental and biological risks associated with nanomaterials [35]. In contrast, microalgae-mediated synthesis exploits natural biological molecules such as proteins, polysaccharides, polyphenols, terpenoids, and pigments that simultaneously act as both reducing agents and stabilizing (end-capping) ligands [20,22]. These natural biological molecules are layered on the surface of the nanoparticles, forming a “biological cap”, which not only provides good colloidal stability to the particles but, more importantly, significantly improves the interface compatibility of the nanoparticles, thereby reducing their direct toxicity to plants and non-target organisms [31].

Beyond reduced chemical toxicity, microalgae-mediated nanoparticles consistently demonstrate high levels of biocompatibility, further supporting their safe deployment in biological systems. Studies using living algal platforms have shown that biosynthesized nanoparticles can coexist with viable cells without disrupting cellular integrity, highlighting favorable nano–bio interactions [152]. Additionally, natural products extracted from microalgae have been demonstrated to reduce nanoparticle-induced oxidative stress when used as surface modifiers or encapsulating agents, effectively attenuating excessive reactive oxygen species generation and moderating nanoparticle reactivity [153].

However, green synthesis should not be viewed as a universal solution for eliminating nanotoxicity. The biological effects of nanoparticles are fundamentally governed by their own physicochemical properties, such as particle size, morphology, surface charge, composition, and applied concentration, which ultimately determine their interactions with living systems. Some studies have shown that nanoparticles synthesized by microalgae can exhibit toxic effects when applied beyond optimal thresholds [154]. For example, at high concentrations or prolonged exposure durations, metal nanoparticles and their released ions (e.g., Ag^+^, Zn^2+^) may inhibit microbial growth, disrupt cellular metabolism, and induce cell death in sensitive organisms [76,146]. Additionally, certain metal oxide nanoparticles, such as CuONPs, can be internalized by microalgae cells (such as *Chlamydomonas reinhardtii*) at low concentrations, damaging the photosynthetic system and inducing excessive ROS production, leading to oxidative stress [155,156]. These outcomes clearly show that the environmental safety of microalgae-derived nanoparticles cannot be taken for granted solely based on green synthesis. Instead, precise dosage optimization, formulation control, and comprehensive ecotoxicological assessments across multiple trophic levels are essential prerequisites for their responsible deployment in agricultural systems.

### 6.2. Effects on Soil Microbial Communities

When nanomaterials enter agricultural soils through methods such as solid fertilization application and irrigation, their potential impact on soil and environmental microorganisms cannot be ignored. Soil microbiotas play fundamental roles in nutrient cycling, organic matter decomposition, soil structure formation, and plant health. While the antimicrobial properties of nanomaterials underpin many of their agricultural benefits, these same properties raise concerns regarding potential unintended effects on non-target beneficial microorganisms. Disruption of key microbial populations could alter soil ecological balance, affect nutrient availability, and compromise long-term soil fertility.

Nanoparticles such as AgNPs and ZnONPs are well documented for their strong antibacterial activity against a wide range of microorganisms [157]. Nevertheless, once introduced into agricultural soils, these nanomaterials may exert non-selective effects, inhibiting or eliminating beneficial microorganisms essential for soil health and crop productivity. These may include nitrogen-fixing bacteria, phosphate-solubilizing bacteria, and plant-promoting rhizosphere bacteria (PGPR), all of which play key roles in nutrient cycling and plant development. For instance, studies have shown that AgNPs and ZnONPs have inhibitory effects on the growth of nitrogen-fixing bacteria, suggesting that they may have a detrimental impact on nitrogen-fixing microorganisms in the soil [158,159]. Such disruptions could ultimately impair soil fertility and destabilize microbial community structure. Therefore, in agricultural applications, it is necessary to assess their long-term and cumulative impacts on key functional microbial groups to ensure that antimicrobial benefits do not compromise soil ecological functions and sustainability.

However, soil microorganisms are not merely passive recipients of nanoparticle exposure and may exhibit adaptive or protective responses. Several studies have demonstrated that certain microorganisms can develop defense mechanisms against nanoparticles. For instance, some bacteria can form physical barriers by secreting extracellular polymers (EPS) that limit direct nanoparticle–cell membrane interactions and thereby reduce their toxicity [154,160]. Building on this, strategies such as immobilizing metal oxide nanoparticles (e.g., ZrO_2_) onto inert matrices have been shown to markedly reduce their toxicity to microorganisms, offering valuable insights for the design of safer nano-enabled agricultural inputs [161].

### 6.3. Carbon Sequestration and Circular Agriculture

Microalgae-mediated nanotechnology offers significant opportunities for integration within circular agricultural systems, particularly in the context of carbon sequestration and resource recycling. Owing to their rapid growth rates, high photosynthetic efficiency, and effective nutrient assimilation, microalgae serve as sustainable biological platforms capable of capturing CO_2_ and converting wastewater into value-added nanomaterials and agricultural inputs.

Microalgae-based nano-synthesis also offers an innovative strategy for treating agricultural and industrial wastewater while simultaneously recovering high-value metals from it. Some industrial wastewater is rich in heavy metals (such as Cu^2+^), which can serve as feedstock for the synthesis of metal nanoparticles by microalgae [50]. *Chlamydomonas reinhardtii* has been shown to convert Cu^2+^ ions into CuNPs while treating copper-containing wastewater [162]. This process combines the bioremediation of harmful pollutants with the green manufacturing of high-value nanomaterials, perfectly embodying the concept of a circular economy. Similarly, microalgae-based treatment of brewery wastewater has been reported to improve water quality while producing algal biomass that can subsequently be valorized for the synthesis of iron nanoparticles (FeNPs), enabling hierarchical resource utilization across multiple processing stages [163].

**Figure 6:**
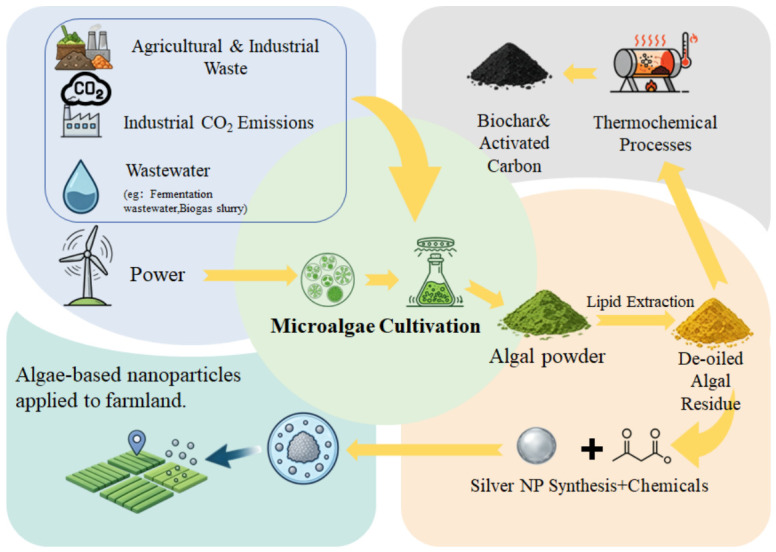
Conceptual model of a circular nano-agriculture system based on microalgae-mediated nanotechnology.

In the microalgal biorefinery process, after the extraction of lipids from microalgal biomass to produce biodiesel, large quantities of defatted algal residue are generated as a by-product. This residual biomass is rich in organic substances such as proteins and carbohydrates, making it a valuable feedstock for subsequent valorization such as the production of bioenergy or bio-based chemicals. For instance, defatted algal biomass and its extracts have been successfully employed for the green synthesis of nanomaterials; silver nanoparticles biosynthesized from lipid-extracted *Acutodesmus dimorphus* exhibited strong antioxidant activity [101]. Figure 6 illustrates a closed-loop nano-agriculture system in which microalgae capture industrial CO_2_ and remediate heavy-metal-contaminated runoff. The harvested biomass is used to biosynthesize metal nanoparticles (e.g., Ag, ZnO, CuO) for sustainable crop production, while residual algal material is converted into biochar and bio-based products for soil improvement, enabling efficient resource recovery and circular agricultural sustainability.

## 7. Challenges, Regulatory Issues, and Public Acceptance

### 7.1. Technical and Scale-Up Challenges

Although microalgae-mediated nanotechnology has shown great potential in sustainable agriculture, its transition from laboratory research to practical field application still faces multiple bottlenecks [164,165]. Firstly, the efficient production and stable supply of microalgae biomass are the primary constraints. Currently, when microalgae cultivation is scaled up from laboratory scale to industrial production, changes in key operational parameters such as light, temperature, and CO_2_ distribution can significantly hamper biomass productivity and cause fluctuations in cellular metabolic profiles. These instabilities can markedly affect both the yield and compositional consistency of the harvested biomass, thereby compromising the uniformity, reproducibility, and continuity of feedstocks required for nanoparticle synthesis [166,167]. Secondly, the large-scale synthesis and quality control of microalgae-based nanomaterials remain insufficiently developed. While microalgae-mediated “green synthesis” offers clear environmental advantages, nanoparticle formation is highly sensitive to multiple biological and process parameters, including algal species selection, extract composition and concentration, incubation time, pH, and temperature. Variations in these factors can lead to significant batch-to-batch differences in nanoparticle size, morphology, surface chemistry, and functionality, making reproducibility and standardization particularly challenging at scale [37,168].

### 7.2. Economic Feasibility

Economic feasibility remains a major barrier to large-scale deployment. When compared with chemically synthesized nanomaterials, microalgae-based nanomaterials have relatively lower yields and more complex separation and purification processes, further increasing production costs. Currently, the production cost of microalgal biomass, particularly commercially cultivated strains such as *Spirulina*, is substantially higher than that of conventional agricultural inputs, limiting its competitiveness for broadacre applications [166]. Finally, the stability, persistence, and interaction mechanisms of microalgae-based nanomaterials in complex field environments, as well as their interactions with soil components (such as organic matter and clay minerals), are still unclear, making their performance in practical applications difficult to predict [164].

### 7.3. Public Acceptance and Regulatory Gaps

For microalgae-mediated nanotechnology to transition effectively into agricultural practice, two critical non-technical barriers must be addressed: regulatory approval and public acceptance [169]. From a regulatory perspective, there is currently a lack of clear, harmonized frameworks governing the oversight of microalgae-derived nanomaterials at the international level. The existing regulatory framework mostly targets traditional chemicals or single-component nanomaterials and is therefore poorly suited to complex products that simultaneously exhibit biological functionality and nanoscale properties. In the EU, the European Food Safety Authority (EFSA) guidance requires nano-specific characterization and risk assessment for food/feed-chain applications, including pesticides, while the regulation on the registration, evaluation, authorization and restriction of chemicals (REACH) introduces nanoform-specific data requirements and Regulation (EU) 2019/1009 governs EU fertilizing products and plant nutrition-efficiency products. FAO/WHO also emphasized risk assessment, life-cycle considerations, and stakeholder communication for nanotechnologies used in agriculture, including agrochemicals [170,171,172]. This creates ambiguity in the classification and assessment of microalgae-derived nanomaterials, particularly for products such as nano-bio-stimulants or nano-pesticides. Key regulatory challenges include defining whether these products should be governed under biopesticide, nano-pesticide, or fertilizer regulations and establishing appropriate methodologies to evaluate their environmental persistence, residue behavior, and potential transmission through the food chain [164,169]. In terms of public acceptance, limited understanding of nanotechnology and its perceived risks may result in societal resistance like that previously encountered with genetically modified organisms. Acceptance of microalgae-derived products as food or feed inputs already varies across regions and cultural contexts, and the introduction of nanoscale terminology may further heighten uncertainty and public concern [166,169]. Additionally, whether microalgae nanomaterials will have negative ecological effects on non-target organisms (such as pollinating insects and soil microbial communities) after field application is also a focus of concern for the public and the scientific community [37,164]. Therefore, establishing a transparent and scientific risk communication mechanism and ethical norms is crucial for enhancing the acceptance of all sectors of society.

## 8. Future Perspectives and Conclusions

Microalgae-mediated nanotechnology is poised to evolve toward greater functional specificity, intelligence, and precision. In the field of intelligent delivery systems, constructing drug-loading systems that respond to environmental signals based on the unique biological structures of microalgae (such as the mesoporous shell walls of diatoms) is an important breakthrough. Recent studies have successfully developed silicified soil microcarriers with dual pH and enzyme response characteristics, enabling the controlled release of pesticides in the midgut environment of small whiteflies (pH > 9). This targeted release strategy significantly reduces pesticide toxicity to non-target organisms while maintaining high pest control efficiency, and surface functionalization further enhances leaf adhesion and wash-off resistance, thereby lowering chemical residues [164]. This paradigm demonstrates that using the natural structures of microalgae to construct “on-demand release” intelligent delivery systems has great potential for improving pesticide utilization and reducing environmental risks [164,173]. This emerging paradigm demonstrates that leveraging the intrinsic biological architectures of microalgae to construct on-demand, stimuli-responsive delivery systems holds considerable promise for enhancing pesticide-use efficiency while minimizing environmental risks [165,174].

In conclusion, microalgae-mediated nanotechnology represents a promising intersection of photosynthetic biotechnology, green nanomaterial synthesis, and sustainable crop management. Microalgae can function not only as biological reducing and stabilizing platforms for nanoparticle production but also as renewable feedstocks that connect carbon capture, wastewater remediation, biomass valorization, and agricultural input development. Current evidence indicates that microalgae-derived nanomaterials have broad potential as bio-nanopesticides, nano-fertilizers, bio-stimulants, and stress-mitigation agents. However, their practical deployment requires a shift from proof-of-concept studies toward mechanism-guided material design, standardized physicochemical characterization, reproducible synthesis protocols, dose–response evaluation, long-term field validation, and life-cycle safety assessment. Future research should place greater emphasis on crop-specific formulations, environmentally responsive delivery systems, safety-by-design strategies, and harmonized regulatory frameworks. With these advances, microalgae-mediated nanotechnology could contribute to reducing agrochemical dependence, improving nutrient-use efficiency, enhancing crop resilience, and promoting circular agricultural production systems.

## Figures and Tables

**Table 1:** Bioactive substances involved in microalgae-mediated nanoparticle synthesis and their primary functions.

Bioactive Substance	Primary Function in Nanoparticle Synthesis	Microalgal Species	Reference
Polysaccharide	Act as reducing agents and stabilizers through abundant functional groups (e.g., hydroxyl, aldehyde, and carboxyl), facilitating metal-ion reduction and steric stabilization of nanoparticles	*Chlorella vulgaris*	[69]
		*Graesiella emersonii*	[1]
		*Spirulina platensis*	[70]
Proteins and Enzymes	Chelate metal ions; catalyze reduction reactions (e.g., nitrate reductase and oxidoreductases); form capping layers that enhance nanoparticle stability	*Chlamydomonas reinhardtii*	[12,16]
		*Macrocyclus pyrifera*	[71]
		*Arthrospira platensis*	[14]
		*Sprulina* sp.	[11,72]
Photosynthetic pigments	Provide high-energy electrons (reduced by light excitation) to restore metal ions. Some pigments themselves possess reducing capabilities.	*Phormidium tenue*	[15]
Fatty acids and their esters	Function mainly as terminating and capping agents, contributing to surface passivation, hydrophobic stabilization, and prevention of aggregation	*Coelastrellia aeroterrestrica*	[27]
		*Isochrysis* sp.	[24]
Extracellular Polymers (EPS)	Provide both reducing functional groups and a protective coating layer; play a dominant role in colloidal stabilization and long-term dispersion stability	*Chlamydomonas reinhardtii*	[73]
		*Arthrospira platensis*	[20]
		*Chlorella* sp.	[73]

**Table 2:** Comparative summary of the antibacterial activity and mechanisms of action of microalgae-derived nanoparticles against major agricultural pathogens.

Pathogen Category	Target Pathogen	Associated Disease	Microalgae Source	Nanoparticle Type	Antimicrobial Activity	Primary Mode of Action	References
Bacteria	*Erwinia amylovora*	Fire blight	*Arthrospira platensis*	AgNPs	Inhibition zone diameter: 17.0 mm; MIC: 0.625 μg/mL	Physical damage: Cell-surface adhesion and penetration leading to membrane rupture and leakage of intracellular contents	[94]
Bacteria	*Erwinia pyrifoliae*	Fire blight	*Cyanothece* sp.	AgNPs	Broad-spectrum antibacterial activity	Physical damage: Size-dependent membrane penetration; smaller particles show higher antibacterial efficacy	[135]
Bacteria	*Xanthomonas citri*	Citrus bacterial canker	*Cyanothece* sp.	AgNPs	Broad-spectrum antibacterial activity	Physical damage: Physical disruption of bacterial membranes; enhanced penetration by small nanoparticles	[135]
Fungi	*Fusarium oxysporum*	Fusarium wilt disease	*Chlorella* K01	Fe_3_O_4_NPs	Inhibit the growth of fungi	Membrane damage and/or indirect induction of plant defense responses	[137]
Fungi	*Fusarium oxysporum*	Fusarium wilt disease	*Desmonostoc alborizicum*	SeNPs	MIC: 10.33 μg/mL (at 10 μg/mL)	ROS generation, lipid peroxidation, DNA damage, and membrane penetration	[138]
Fungi	*Alternaria alternata*	Black spot	*Desmonostoc alborizicum*	SeNPs	MIC: 7.66 μg/mL (most sensitive)	Enhanced oxidative stress leading to structural and genomic damage	[137]
Fungi	*Pythium ultimum*	Botrytis infection	*Desmonostoc alborizicum*	SeNPs	MIC: 11.33 μg/mL (the most resistant)	ROS-mediated membrane and cellular damage	[141]
Fungi	*Rhizoctonia solani*	Damping off	*Chlorella* K01	Fe_3_O_4_NPs	ZOI: ~10–25 mm	Iron-mediated surface reactivity and disruption of fungal cell wall integrity	[137]
Fungi	*Phythium* sp.	Botrytis infection	*Chlorella* K01	Fe_3_O_4_NPs	ZOI: ~10–25 mm	Cell wall and membrane damage via iron-associated surface activity integrity of the fungal cell wall/membrane.	[141]
Fungi	*Macrophomina phaseolina*	Bark rot disease	*Ulva fasciata*	CH-Mg-alg nano-composite	In vitro inhibition rate: 88.9%; Disease severity reduction: 56.4% → 23.8%	Synergistic interaction of chitosan and MgNPs causing hyphal deformation and membrane damage	[139]
Fungi	*Aspergillus flavus*	Mycotoxin disease	*Nodosilinea nodulosa*	Co_3_O_4_NPs	ZOI: 5 mm (200 μg/mL)	ROS production resulting in oxidative cellular damage	[142]
Fungi	*Fusarium oxysporum*	Fusarium wilt disease	*Nodosilinea nodulosa*	Co_3_O_4_NPs	ZOI: 7 mm (200 μg/mL)	Oxidative stress-induced membrane disruption	[142]
Oomycetes	*Phytophthora infestans*	Tomato late blight	*Eucheuma* sp.	AgNPs	Inhibit the growth of oomycetes	Membrane damage via physical and chemical nanoparticle interactions	[143]
Virus	Tobacco mosaic virus (TMV)	Mosaic disease	Fucoidan (brown algae)	AgNPs/AuNPs	Significantly inhibit the virus in vitro	Capsid interaction and blockage of viral entry or intracellular decoy mechanisms	[140]

## Data Availability

No new data were created or analyzed in this study.
